# Acute blood glucose fluctuation enhances rat aorta endothelial cell apoptosis, oxidative stress and pro-inflammatory cytokine expression in vivo

**DOI:** 10.1186/s12933-016-0427-0

**Published:** 2016-08-05

**Authors:** Na Wu, Haitao Shen, Henan Liu, Yanjun Wang, Yu Bai, Ping Han

**Affiliations:** 1 Department of Endocrinology, Shengjing Hospital of China Medical University, Shenyang, 110004 China; 2 Department of Emergency, Shengjing Hospital of China Medical University, Shenyang, 110004 China; 3 Department of Ophthalmology, Shengjing Hospital of China Medical University, Shenyang, 110004 China

**Keywords:** Acute blood glucose fluctuation, Endothelial cells, Apoptosis, Oxidative stress, Inflammation

## Abstract

**Background:**

Complications of diabetes mellitus (DM) are related not only to elevated plasma glucose, but also plasma glucose fluctuations. However, the specific mechanism underlying the role of plasma glucose fluctuation in the pathogenesis of DM complications remains poorly understood. In the present study, the influence of acute fluctuant hyperglycemia and persistent hyperglycemia on vascular endothelial cell apoptosis, function, oxidative stress and inflammation was examined in vivo.

**Methods:**

Rats were assigned to three different groups (n = 10/group) that received 48-h infusions of saline (SAL group), continuous 50 % glucose (constant high glucose group [CHG]), or intermittent 50 % glucose (acute blood glucose fluctuation group [AFG]). Plasma 8-isoprostaglandin, interleukin-6 (IL-6), tumor necrosis factor-α (TNF-α) and intercellular adhesion molecule-1 (ICAM-1) levels were quantified by using enzyme-linked immunosorbent assay (ELISA) commercial kits. Plasma insulin levels were measured by radioimmunoassays (RIAs) using kits. The aortic segment was collected. The levels of malondialdehyde (MDA) and activity of glutathione peroxidase (GSH-PX) were measured in endothelial homogenates prepared from endothelial cells harvested from the aorta using colorimetric kits. Apoptosis of vascular endothelial cells was determined with terminal deoxynucleotidyl transferase dUTP nick end labeling (TUNEL). Endothelial dysfunction was assessed by isometric tension recording to evaluate the endothelial function. The expression of B cell lymphoma-2 (Bcl-2), Bcl-2 Associated X protein (Bax), pro caspase-3, caspase-3 p17, 3-nitrotyrosine (3-NT) and p47phox protein in rat aortic endothelial cells were tested with Western blot analysis. Endothelial cells reactive oxygen species (ROS) formation was determined using dihydroethidium-dependent fluorescence microtopography in aortic cryo-sections. Expression of IL-6, TNF-α and ICAM-1 mRNAs in vascular endothelial cells were determined by real-time quantitative PCR.

**Results:**

Endothelial cells apoptosis and dysfunction were observed significantly in the aortas of the AFG group (P < 0.05). The AFG had reduced Bcl-2 and pro caspase-3 levels and enhanced Bax mitochondrial translocation and caspase-3 p17 protein levels in comparison with the CHG group (P < 0.05). Both AFG and CHG induced β-cell dysfunction and insulin resistance (P < 0.05). AFG increased MDA and 8-isoprostaglandin levels in plasma, oxidative stress in vascular endothelial cells, and inflammatory cytokines in plasma and vascular endothelial cells (P < 0.05).

**Conclusion:**

Acute glucose fluctuation may cause significant oxidative stress and inflammation in endothelial cells, increase the adhesion of monocytes to endothelial cells, and elevate endothelial cell apoptosis, resulting in severe cardiovascular injury.

## Background

In recent years, studies have indicated that the occurrence and development of diabetes mellitus (DM) complications are closely related to not only elevated plasma glucose (PG), but also fluctuations in PG [1]. Compared to persistent hyperglycemia, fluctuant hyperglycemia has more potential to increase microvascular lesions and the risk of cardiovascular death [2–4], but the specific mechanism is still unclear. Blood glucose fluctuation exists not only in diabetes, but also in individuals with normal metabolic function. In the condition of stress,the glucose levels of those with normal metabolic function may be very high. In this case given hypoglycemic therapy, there may be glucose decreases sharply in the hypoglycemic process, leading to acute glucose fluctuations. We had successfully established acute blood glucose fluctuation model before [5, 6]. Evidence indicates that oxidative stress and inflammation are the major mechanisms underlying the pathogenesis of DM complications. The vicious cycle of oxidative stress and inflammation may damage cells, and increasing evidence suggests that acute PG fluctuation may induce oxidative stress in vivo [5].

Endothelial dysfunction is a key pathophysiological step in the early stage of vascular DM complications [7–9]. Previous studies have demonstrated that vascular dysfunction in the setting of diabetes is associated with increased vascular oxidative stress and low-grade inflammation [10–12]. Early in vitro studies showed that fluctuant hyperglycemia could increase the apoptosis of human umbilical vein endothelial cells [13, 14] and induce inflammation [15]. In recent years, in vitro studies have also indicated that fluctuant hyperglycemia may increase apoptosis, oxidative stress [16] and inflammation [17, 18] of human coronary artery endothelial cells (HCAECs). Mita et al. [19] demonstrated that swings in blood glucose levels accelerated atherogenesis in apolipoprotein E-deficient mice. In Mita T’s study, apolipoproteine-deficient mice fed maltose twice daily were used as a model of repetitive postprandial glucose spikes, which simulated chronic hyperglycemia in vivo. Blood glucose fluctuation exists not only in diabetes, but also in individuals with normal metabolic function. In the condition of stress,the glucose levels of those with normal metabolic function may be very high. In this case given hypoglycemic therapy, there may be glucose decreases sharply in the hypoglycemic process, leading to acute glucose fluctuations. To date, few studies have been conducted to investigate the influence of acute fluctuant hyperglycemia on endothelial cells in vivo. Our group had successfully established the acute fluctuant hyperglycemia animal model [5, 6], which was employed to investigate the effects of fluctuant hyperglycemia on endothelial cells.

In this study, glucose was intravenously injected into rats to establish animal models of acute fluctuant hyperglycemia and persistent hyperglycemia with the objective of exploring their influence on apoptosis, oxidative stress and inflammation of endothelial cells in vivo.

## Methods

### Animals

Normal male Wistar rats weighing 250–350 g (8–10 weeks old) were provided by the Medical Animal Center, Shengjing Hospital of the China Medical University (Shenyang, China). The rats were subjected to a standard 12:12-h light–dark cycle and were given free access to regular chow and water. All animal experiments were performed with approval from the animal ethics committee of Shengjing Hospital of China Medical University (2016PS026 K). All experiments conformed to guidelines for ethical conduct in the care and use of animals. Every effort was made to minimize stress to the animals.

### Animal surgeries

Indwelling catheters (PE-50; Cay Adams, Boston, MA, USA), each extended with a 3-cm segment of silastic tubing with an internal diameter 0.02 in (Care Express Products, Inc., Cary, IL, USA), were inserted into the internal jugular vein of the rats for infusion and the carotid artery for blood sampling as described previously [20]. After surgery, the rats had 3–4 days of recovery before intravenous infusion.

### Intravenous infusions

**Fig. 1:**
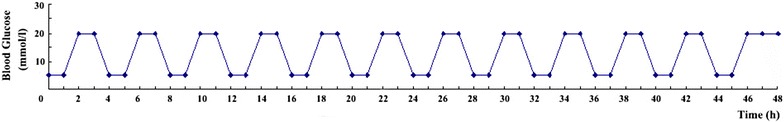
Acute blood glucose fluctuation model. Blood glucose alternated between 5.5 ± 0.5 and 20 ± 0.5 mmol/l

Food was withdrawn 12–14 h before each infusion. The rats were assigned to three different groups (n = 10/group), each of which underwent 48-h infusions with (1) saline (5.5 mL/min; SAL group); (2) continual 50 % glucose (blood glucose was monitored regularly to keep the glucose concentration at 20 ± 0.5 mmol/l, constant high glucose [CHG] group); or (3) intermittent 50 % glucose (Every 15 min, we measured the blood glucose level, and adjusted the glucose infusion rate according to the blood glucose monitoring. The blood glucose level alternated between 5.5 ± 0.5 and 20 ± 0.5 mmol/L. That is to say, control the glucose level at about 5.5 mmol/l for 1 h, and then at 20 mmol/l for 1 h, so the cycle. Acute blood glucose fluctuation [AFG] group) (Fig. 1). The procedures for infusion were followed as previously described [5].

### Plasma assays

Glucose concentrations in plasma were measured with a Beckman Glucose Analyzer II (Beckman, Fullerton, Calif., USA). At the beginning and the end of the 48 h infusion, plasma insulin levels were measured by radioimmunoassays (RIAs) using kits specific for rat (Beijing Furui Biological Engineering Co, China). With plasma insulin and glucose levels, insulin resistance was estimated as homeostasis model of assessment for insulin resistence index (HOMA-IR) using HOMA Calculator v2.2.3 offered by University of Oxford. Insulin secretion (β cell function) was obtained as HOMA-β % using HOMA Calculator. Circulating oxidative stress, measured as the plasma concentration of 8-isoprostaglandin, was analyzed using an enzyme-linked immunosorbent assay (ELISA) commercial kits (Uscnlife, Missouri, TX, USA). The plasma levels of malondialdehyde (MDA) were measured using colorimetric kits (Nanjing Jiancheng Institute of Bio-engineering, China). Plasma interleukin-6 (IL-6), tumor necrosis factor-α (TNF-α) and intercellular adhesion molecule-1 (ICAM-1) levels were quantified by using commercially available ELISA kits (Uscnlife, Missouri, TX, USA), according to the manufacturer’s instructions.

### Tissue collection and preparation of endothelial cell homogenates

After 48 h of infusion, rats were anesthetized with an intraperitoneal injection of chloral hydrate (400 mg/kg). After blood collection, the animals were perfused with 100 mL of normal saline. Then, an aortic segment was collected to detect apoptosis, reactive oxygen species (ROS) formation and vascular function. Another aortic segment was collected, and vascular endothelial cells were harvested followed by extraction of protein for western blotting assay. The procedures for endothelial cell homogenate sample preparation were followed as previously described [21]. Briefly, after opening the aorta longitudinally and rinsing it with PBS, the inner surface was gently scraped with the tip of a scalpel to selectively isolate endothelial cells. The endothelial scrapings from each rat aorta were sonicated in 200 μl of ice-cold homogenizing buffer. The aortic endothelial homogenate was clarified by centrifugation (13,800×*g* for 10 min at 4 °C), snap-frozen and stored at −80 °C for Western blot analysis. Protein concentrations were determined by the Coomassie brilliant blue G-250 dye-binding method.

### Terminal deoxynucleotidyl transferase dUTP nick end labeling (TUNEL) assay

Paraffin-embedded aorta sections were processed for a terminal deoxynucleotidyl transferase dUTP nick end labeling assay with kits from Roche Company (Mannheim, Germany) according to the manufacturer’s instructions. Sections were deparaffinized and rehydrated. After washing with PBS three times, all sections were incubated for 8 min in freshly prepared 0.1 % Triton X-100 permeabilization solution with 0.1 % citrate buffer and then washed with PBS. A TUNEL TdT enzyme reaction mixture (50 µL) was added to each sample and incubated for 1 h in a humidifying chamber at 37 °C. Slides were then washed and observed under a fluorescence microscope.

### Endothelial function studies

We detected acetylcholine (Ach)-dependent vasodilatation by isometric tension recording to evaluate the endothelial function. Vasodilator responses to the endothelium-dependent vasodilator Ach were assessed in organ chambers by isometric tension studies, preconstricted with phenylephrine (PheE), as described previously, as previously described [22].

### Western blot analysis of B-cell lymphoma-2 (Bcl-2), Bcl-2 associated X protein (Bax), pro caspase-3, caspase-3 p17, 3-nitrotyrosine (3-NT) and p47phox protein expression

Endothelial cell homogenates containing equal amounts of protein were separated using SDS polyacrylamide gel electrophoresis (SDS-PAGE) and were transferred onto polyvinylidene fluoride (PVDF) membranes. To investigate the membrane association of soluble NADPH-oxidase subunits p47phox, the lysates were separated into cytosolic and membrane fractions by ultracentrifugation (100,000×*g* for 1 h at 4 °C). The membranes were blocked in Tris-buffered saline-Tween (TBST) containing 5 % non-fat dried milk (pH 7.4) for 2 h at room temperature and then incubated with one of the following primary antibodies: monoclonal mouse Bax (1:200, Santa Cruz Biotechnology, Santa Cruz, CA, USA), monoclonal mouse Bcl-2 (1:1000, Santa Cruz Biotechnology), monoclonal mouse caspase-3 (1:200, Santa Cruz Biotechnology), polyclonal goat caspase-3 p17, 1:1000, Santa Cruz Biotechnology), 3-NT (1:1400, Abcam, Cambridge, MA, USA),monoclonal mouse p47phox(1:500, Santa Cruz Biotechnology), monoclonal mouse GAPDH (1:5000, Abcam), monoclonal mouse COX-IV (1 μg/ml, Abcam), monoclonal rabbit Na/K ATPase (1:100000, Abcam) and monoclonal mouse β-actin (1:1000, Santa Cruz Biotechnology). After the membranes were washed with TBST, incubated with a horseradish peroxidase-conjugated secondary antibody for 2 h at room temperature, the bands were exposed using an enhanced chemiluminescence (ECL) kit (Pierce Biotechnology, Rockford, IL, USA). Protein bands were visualized using ChemDocTM XRS with Quantity OneTM software (BioRad, Hercules, CA, USA). Blots were repeated at least three times for every condition. After development, the band intensities were quantified using Image-pro Plus 6.0 analysis software. The relative protein levels were calculated based on β-actin or GAPDH or Na/K ATPase as the loading control.

### MDA level, glutathione peroxidase (GSH-PX) activity and ROS formation analysis

The levels of MDA and activity of GSH-PX in the endothelial cells were measured using colorimetric kits (Nanjing Jiancheng Institute of Bio-engineering, Nanjing, China). Endothelial cells ROS formation was determined using dihydroethidium (DHE, Santa Cruz Biotechnology)-dependent fluorescence microtopography in aortic cryo-sections. Briefly, the sections were immersed in 10 μmol/L dihydroethidium (in PBS solution) in a humidified environment at room temperature for 30 min. Dihydroethidium is oxidized by superoxide to form ethidium, which binds to DNA in the nucleus and emits red fluorescence. After washing with PBS three times, the sections were cover-slipped and then visualized under a fluorescence microscope (BX41, Olympus, Tokyo, Japan). Intensity of the DHE oxidation products’ fluorescence was evaluated by densitometry.

### Real-time quantitative PCR analysis

Total RNA was extracted from the endothelial cell homogenates with TRIzol Reagent (Takara, Shiga, Japan). The quantity and quality of the total RNA were determined by spectrophotometry. Then, 1 µg of total RNA was reverse-transcribed to cDNA using the Reverse Transcription System (Takara). Quantitative PCR analysis was carried out using an ABI Prism 7500 Sequence Detection System (Applied Biosystems, Foster City, CA, USA) with SYBR green PCR reagents (Takara). The primers for IL-6, TNF-α, ICAM-1 and β-actin were as follows: IL-6 (sense 5′-TCGAGCCCACCGGGAACGAA-3′; antisense 5′-GCAACTGGACCGAAGGCGCT-3′); TNF-α (sense 5′-CGAGTCTGGGCAGGTCTACTTT-3′; antisense 5′-AGAGGTTGAGGGTGTCTGAAGG-3′); ICAM-1 (sense 5′-CCTTCCTCACCGTGTACTGG-3′; antisense 5′-AGCGTAGGGTAAGGTTCTTGC-3′); β-actin (sense 5′-GGAGATTACTGCCCTGGCTCCTA; antisense 5′-GACTCATCGTACTCCTGCTTGCTG). Relative expression was normalized with β-actin.

### Statistical analysis

Data are expressed as mean ± standard deviation (SD). One-way analysis of variance (ANOVA) with a Tukey’s post hoc test was performed to detect differences between the groups. Analyses of all data were performed using the SPSS software (version 13.0; SPSS, Chicago, IL, USA). P < 0.05 were considered statistically significant.

## Results

### An acute blood glucose fluctuation model was successfully established

In the AFG group, PG fluctuated between 5.5 and 20 mmol/L. PG was maintained at 5.5 mmol/L for about 1 h and then at 20 mmol/L for another 1 h over a period of 48 h (Fig. 1).

### Insulin secretion and insulin resistance

**Fig. 2:**
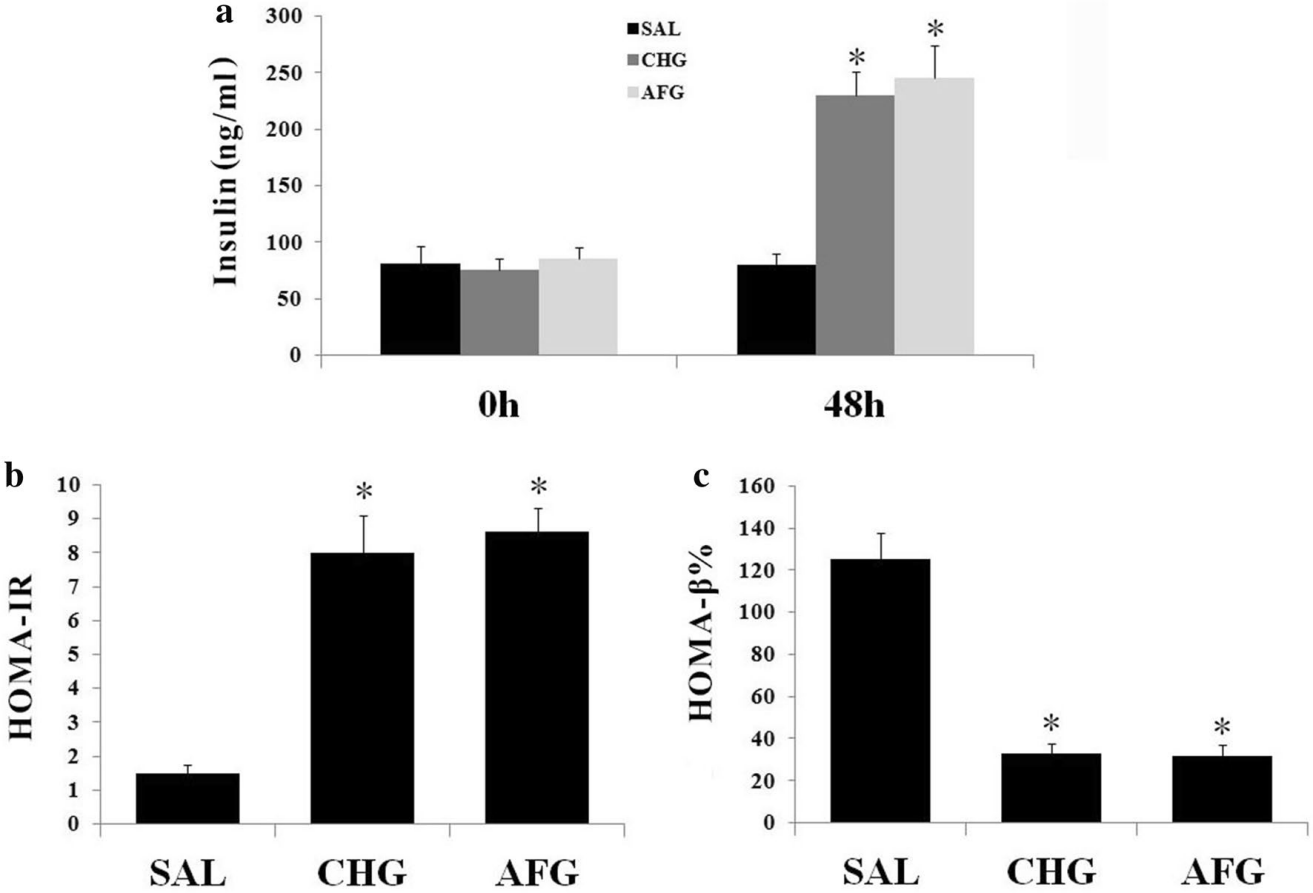
Insulin secretion and insulin resistance. **a** Plasma insulin levels at the beginning and the end of the infusion. **b** Insulin resistance was estimated as HOMA-IR. **c** β cell function was estimated as HOMA-β %. SAL (SAL group rats underwent 48 h infusions with saline); CHG (CHG group rats underwent 48 h infusions with 50 % glucose continually to keep the glucose concentration at 20 ± 0.5 mmol/l); AFG (AFG group rats underwent 48 h infusions with 50 % glucose intermittently so that blood glucose alternated between 5.5 ± 0.5 and 20 ± 0.5 mmol/l). Data are mean ± SD. n = 10/group. *P < 0.05 vs SAL

At the end of the 48 h infusion, insulin levels were significantly increased in the AFG and CHG groups in comparison with the SAL group (P < 0.05; Fig. 2a). The AFG group had higher insulin levels than the CHG group, but the difference was not statistically significant (P > 0.05; Fig. 2a). Insulin resistance, estimated as HOMA-IR, was higher in the AFG and CHG groups than that in the SAL group (P < 0.05; Fig. 2b). In contrast,β cell function, estimated as HOMA-β % was lower in the AFG and CHG groups than that in the SAL group (P < 0.05; Fig. 2c). But neither the HOMA-IR nor HOMA-β % showed significant difference between the AFG and CHG group.

### Apoptosis of vascular endothelial cells

**Fig. 3:**
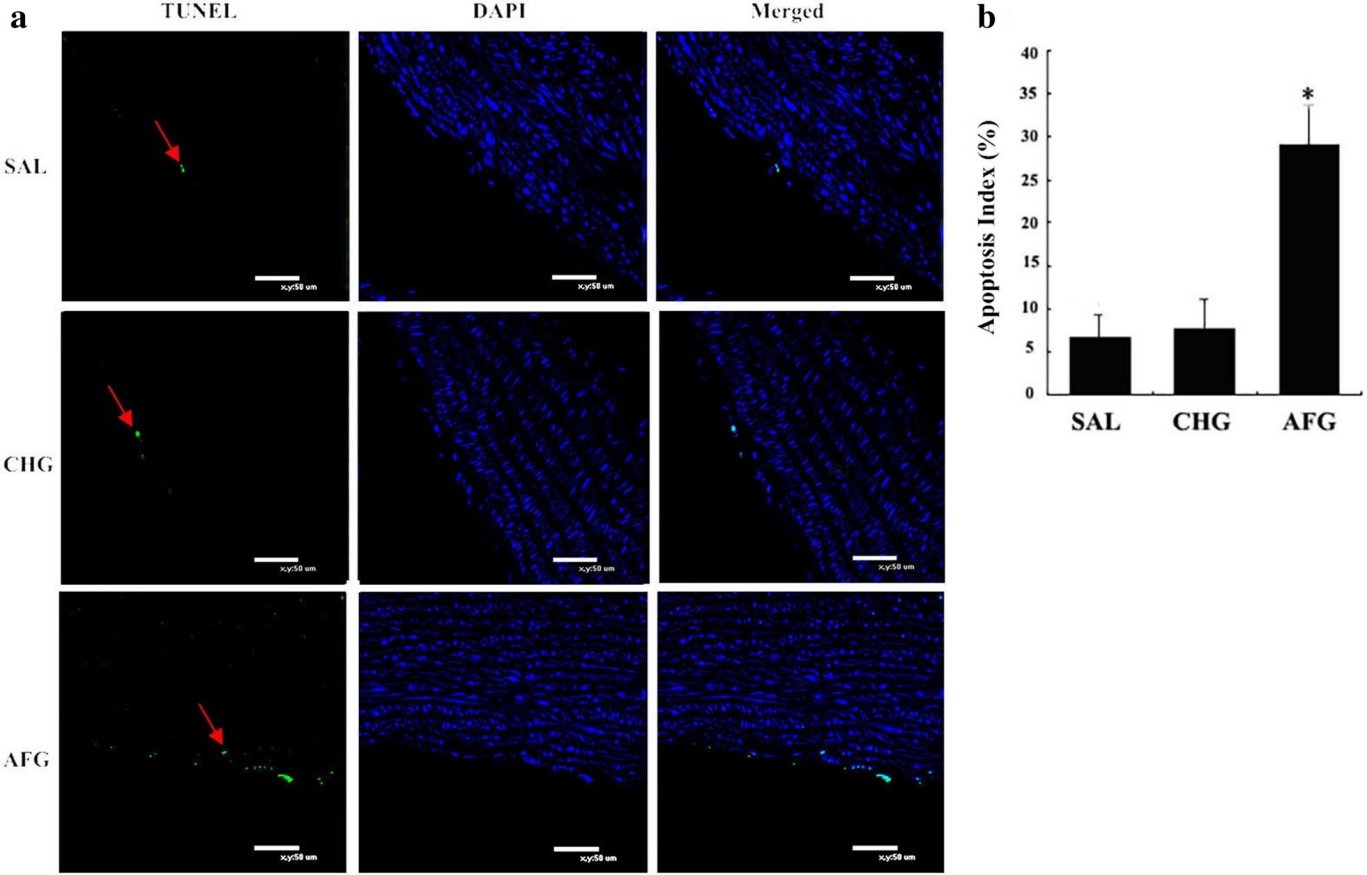
Apoptosis of vascular endothelial cells. TUNEL: bodies were stained with FITC-12-dUTP; DAPI: cell nucleus were stained with DAPI; merged:merge two pictures before. The *red arrow* showed the apoptotic endothelial cells. SAL (SAL group rats underwent 48 h infusions with saline); CHG (CHG group rats underwent 48 h infusions with 50 % glucose continually to keep the glucose concentration at 20 ± 0.5 mmol/l); AFG (AFG group rats underwent 48 h infusions with 50 % glucose intermittently so that blood glucose alternated between 5.5 ± 0.5 and 20 ± 0.5 mmol/l). Data are mean ± SD. n = 10/group. *P < 0.05 vs SAL; ^#^P < 0.05 vs CHG

The apoptosis of aorta vascular endothelial cells was detected using the TUNEL assay. In the AFG group, the apoptosis index was higher than the other two groups. But there is no differences between SAL and CHG group (Fig. 3).

### Endothelial function

**Fig. 4:**
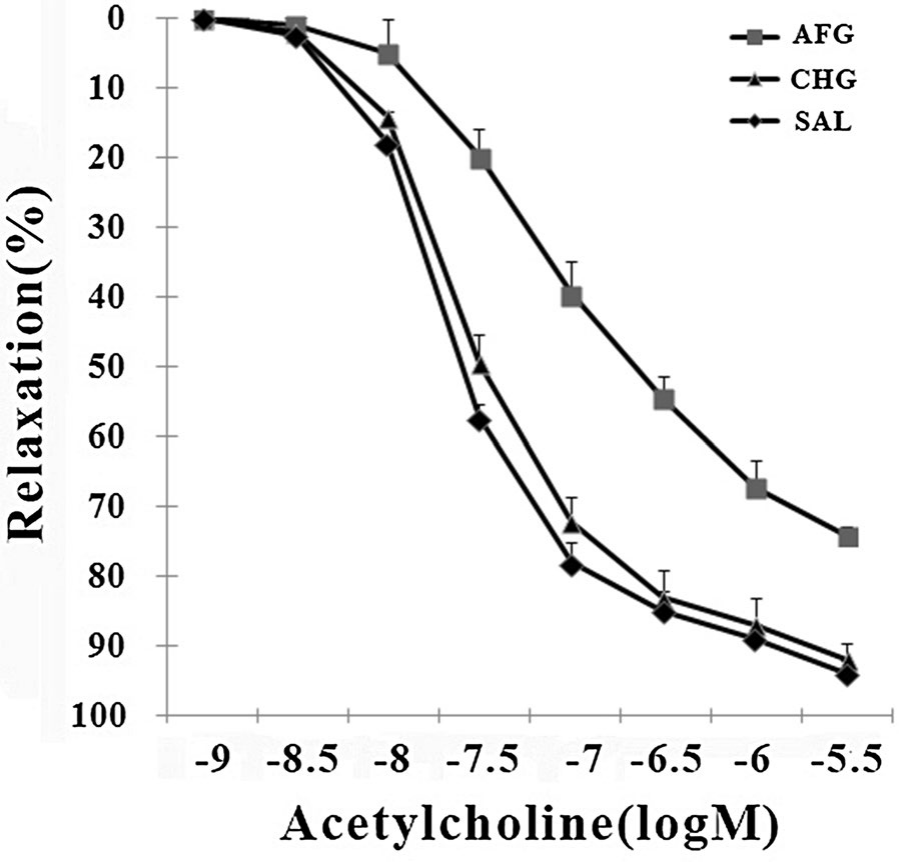
Endothelium-dependent vascular relaxation by the vasodilators acetylcholine (ACh). SAL (SAL group rats underwent 48 h infusions with saline); CHG (CHG group rats underwent 48 h infusions with 50 % glucose continually to keep the glucose concentration at 20 ± 0.5 mmol/l); AFG (AFG group rats underwent 48 h infusions with 50 % glucose intermittently so that blood glucose alternated between 5.5 ± 0.5 and 20 ± 0.5 mmol/l). Data are mean ± SD. n = 10/group

As shown in Fig. 4, AFG caused endothelial dysfunction which resulted in a significant right-shift of the acetylcholine concentration–relaxation curve as observed by isometric tension studies (P < 0.05). Vasodilator potency and efficacy were shown in Table 1. Potency is −log EC50 and EC50 is the concentration that induces half-maximal relaxation with respect to the maximal relaxation achieved with the highest concentration of the vasodilator used. Efficacy is defined as maximal relaxation obtained with the highest concentration of the vasodilator used. Endothelial function in the CHG group is worse than in the CHG group, but the difference was not statistically significant (P < 0.05; Fig. 4; Table 1).

**Table 1:** Potencies and efficacies of the endothelium-dependent vasodilator acetylcholine (Ach) in rat aorta

Parameter	SAL	CHG	AFG
Ach potency (−log EC_50_)	7.9 ± 0.05	7.8 ± 0.06	7.2 ± 0.08*
Ach efficacy (max, relaxation, %)	98 ± 1.2	96 ± 2.4	75 ± 1.8*

SAL (SAL group rats underwent 48 h infusions with saline); CHG (CHG group rats underwent 48 h infusions with 50 % glucose continually to keep the glucose concentration at 20 ± 0.5 mmol/l); AFG (AFG group rats underwent 48 h infusions with 50 % glucose intermittently so that blood glucose alternated between 5.5 ± 0.5 and 20 ± 0.5 mmol/l). Data are mean ± SD. n = 10/group. * P < 0.05 vs SAL

### Expression of Bax, Bcl-2, pro caspase-3 and caspase-3 p17 proteins

**Fig. 5:**
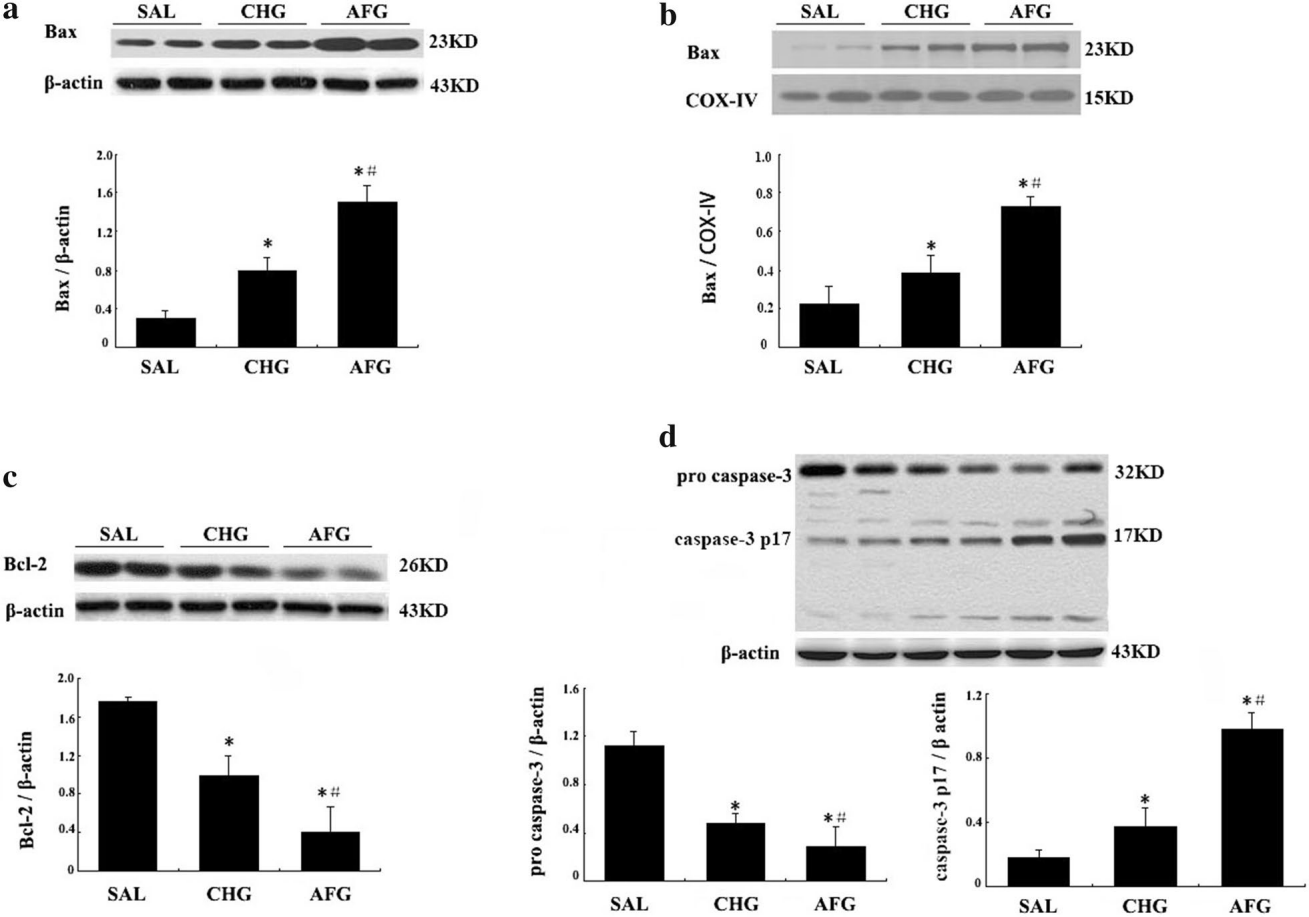
Western blot analysis of Bax, Bcl-2, pro caspase-3 and caspase-3 p17 proteins in vascular endothelial cells. **a** Total bax protein expression in vascular endothelial cells. **b** Mitochondrial Bax protein expression in vascular endothelial cells. **c** Bcl-2 expression in vascular endothelial cells. **d** Pro caspase-3 and caspase-3 p17 expression in vascular endothelial cells. SAL (SAL group rats underwent 48 h infusions with saline); CHG (CHG group rats underwent 48 h infusions with 50 % glucose continually to keep the glucose concentration at 20 ± 0.5 mmol/l); AFG (AFG group rats underwent 48 h infusions with 50 % glucose intermittently so that blood glucose alternated between 5.5 ± 0.5 and 20 ± 0.5 mmol/l). β-Actin antibodies were used as loading controls. A representative western blot is shown. Data are mean ± SD. n = 10/group. *P < 0.05 vs SAL; ^#^P < 0.05 vs CHG

As shown in Fig. 5, total Bax, mitochondrial Bax and caspase-3 p17 protein levels in the CHG and AFG group were all significantly increased compared with the SAL group (P < 0.05; Fig. 5a, b, d). Furthermore, the expression of total Bax, mitochondrial Bax and caspase-3 p17 in the AFG group was higher than that in the CHG group (P < 0.05; Fig. 5a, b, d). In addition, both constant high glucose and acute blood glucose fluctuation decreased the levels of Bcl-2 and pro caspase-3 protein when compared with the SAL group, and the AFG group had significantly reduced Bcl-2 and pro caspase-3 levels in comparison with the CHG group (both P < 0.05; Fig. 5c, d respectively).

### Circulating 8-isoprostaglandin and MDA levels

**Fig. 6:**
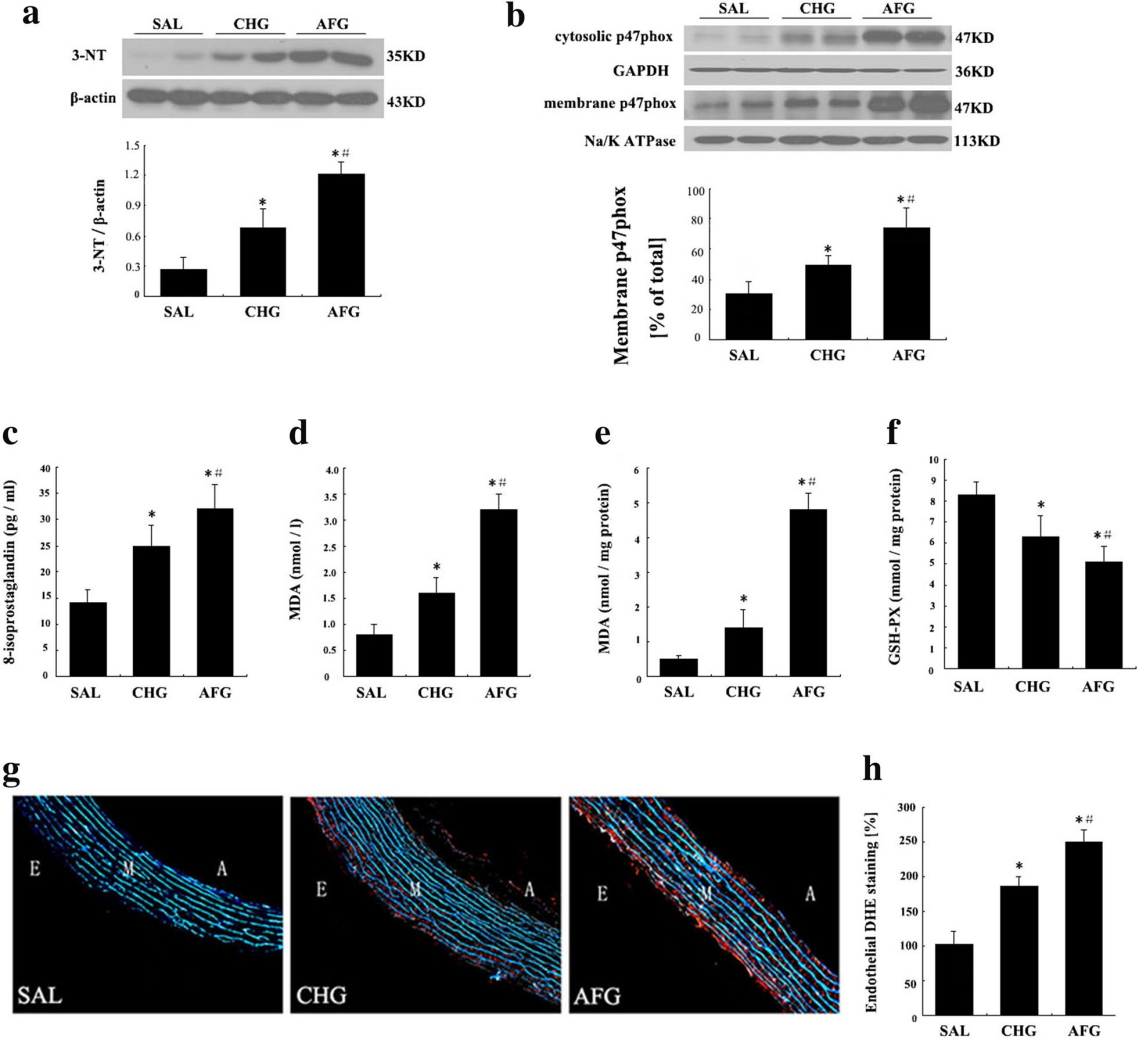
Oxidative stress markers in plasma and vascular endothelial cells. **a** 3-nitrotyrosine (3-NT) expression in vascular endothelial cells. **b** P47phox expression in vascular endothelial cells. **c** 8-isoprostaglandin levels in plasma. **d** Malondialdehyde (MDA) levels in plasma. **e** MDA levels in vascular endothelial cells. **f** Glutathione peroxidase (GSH-PX) activities in vascular endothelial cells. SAL (SAL group rats underwent 48 h infusions with saline); **g** Dihydroethidium (DHE)-fluorescence microtopography was used to assess the effects of glucose treatment on aortic reactive oxygen species (ROS) production. Representative microscope images are shown. *Red fluorescence* indicates ROS formation whereas *green fluorescence* represents basal laminae autofluorescence. **h** Densitometric quantification of the DHE-derived ROS signal in the endothelium of vessels. CHG (CHG group rats underwent 48 h infusions with 50 % glucose continually to keep the glucose concentration at 20 ± 0.5 mmol/l); AFG (AFG group rats underwent 48 h infusions with 50 % glucose intermittently so that blood glucose alternated between 5.5 ± 0.5 and 20 ± 0.5 mmol/l). Data are mean ± SD. n = 10/group. *P < 0.05 vs SAL; ^#^P < 0.05 vs CHG

Circulating oxidative stress in this study was measured as the concentration of 8-isoprostaglandin and MDA. 8-isoprostaglandin is an in vivo oxidative stress marker, which is produced in a non-cyclooxygenase-dependent manner involving lipid peroxidation [23]. MDA, a marker of lipid peroxidation, was also measured. As shown in Fig. 6c and d, plasma 8-isoprostaglandin and MDA levels were significantly increased in the AFG and CHG groups in comparison with the SAL group (P < 0.05). The levels of these two oxidative stress markers were also significantly higher in the AFG group than those in the CHG group (P < 0.05; Fig. 6c, d respectively).

### P47phox and 3-NT protein levels, MDA levels, GSH-PX activities, and ROS formation in vascular endothelial cells

As shown in Fig. 6a, 3-NT protein levels in the CHG and AFG group were significantly increased compared with the SAL group, and the levels in the AFG group were higher than that in the CHG group (P < 0.05; Fig. 6a). Both the cytosolic and the membrane p47phox levels in the CHG and AFG group were significantly increased compared with the SAL group. Most importantly, the ratio of membrane-bound to cytosolic portion of p47phox was also increased in high glucose groups. In addition, the ratio was higher in the AFG group than that in the CHG group (P < 0.05; Fig. 6b). As shown in Fig. 6e, the MDA levels in vascular endothelial cells were significantly increased in the AFG and CHG groups as compared with the SAL group (P < 0.05). Moreover, the AFG group had higher MDA levels than the CHG group (P < 0.05). On the contrary, the GSH-PX activities in vascular endothelial cells were decreased in the fluctuating condition compared with rats exposed to continually high glucose (P < 0.05, Fig. 6f). Furthermore, the GSH-PX activities in the CHG and AFG groups were lower than that of the SAL group (P < 0.05, Fig. 6f). Aortic ROS formation was detected in cryo-sections using DHE dependent fluorescent micro-topography (Fig. 6g), which was quantified by densitometry. Densitometric quantification of the DHE-derived ROS signal in the endothelium of vessels was shown in Fig. 4h. There was a significant increase in ROS formation in the endothelium of vessels from the CHG and AFG rats. And the levels of ROS formation were significantly higher in the AFG group than those in the CHG group (P < 0.05; Fig. 6h).

### Plasma inflammatory cytokine concentrations

**Fig. 7:**
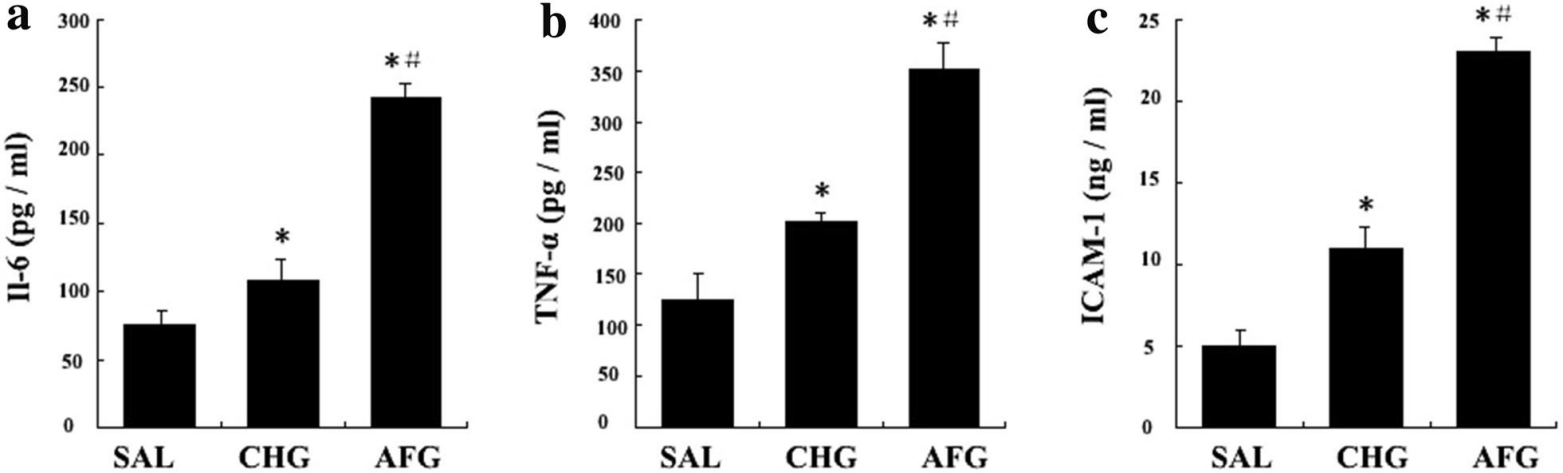
ELISA analysis of IL-6, TNF-α and ICAM-1 in plasma. **a** The Circulating levels of Interleukin-6 (IL-6). **b** The Circulating levels of Tumor necrosis factor-α (TNF-α). **c** The Circulating levels of Intercellular adhesion molecule-1(ICAM-1). SAL (SAL group rats underwent 48 h infusions with saline); CHG (CHG group rats underwent 48 h infusions with 50 % glucose continually to keep the glucose concentration at 20 ± 0.5 mmol/l); AFG (AFG group rats underwent 48 h infusions with 50 % glucose intermittently so that blood glucose alternated between 5.5 ± 0.5 and 20 ± 0.5 mmol/l). Data are mean ± SD. n = 10/group. *P < 0.05 vs SAL; ^#^P < 0.05 vs CHG

The circulating levels of IL-6, TNF-α, and ICAM-1 increased significantly in the CHG and AFG groups compared with the SAL group (P < 0.05, Fig. 7). In addition, the concentrations in the AFG group were higher than that of the CHG group (P < 0.05, Fig. 7).

### IL-6, TNF-α and ICAM-1 mRNA expression in vascular endothelial cells

**Fig. 8:**
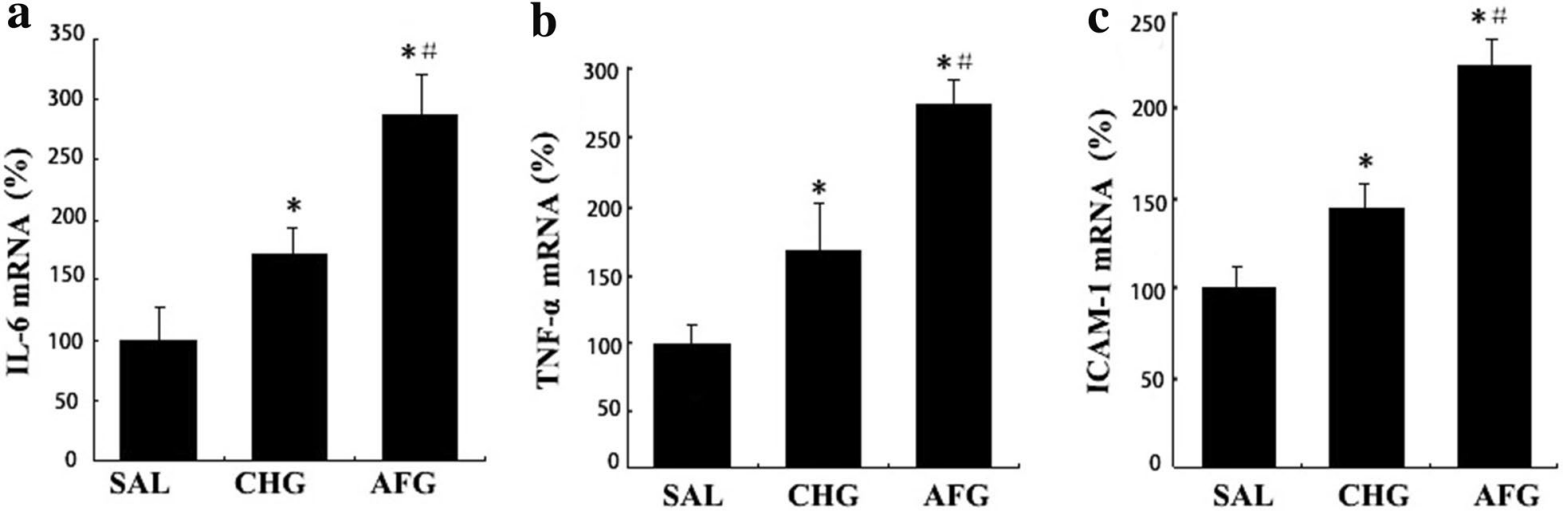
Expression of IL-6, TNF-α and ICAM-1 mRNAs in vascular endothelial cells. **a** The mRNA expressions of Interleukin-6 (IL-6) in vascular endothelial cells. **b** The mRNA expressions of tumor necrosis factor-α (TNF-α) in vascular endothelial cells. **c** The mRNA expressions of intercellular adhesion molecule-1(ICAM-1) in vascular endothelial cells.CHG (CHG group rats underwent 48 h infusions with 50 % glucose continually to keep the glucose concentration at 20 ± 0.5 mmol/l); AFG (AFG group rats underwent 48 h infusions with 50 % glucose intermittently so that blood glucose alternated between 5.5 ± 0.5 and 20 ± 0.5 mmol/l). Data are mean ± SD. n = 10/group. *P < 0.05 vs SAL; ^#^P < 0.05 vs CHG

In the CHG and AFG groups, IL-6, TNF-α and ICAM-1 mRNA expression was significantly higher than the SAL group (P < 0.05, Fig. 8). The mRNA expression of all three cytokines was significantly higher in the AFG group compared to the CHG group (P < 0.05, Fig. 8).

## Discussion

The incidence of cardiovascular diseases in DM patients is significantly higher than in non-DM patients [24]. Vascular endothelial cell injury and dysfunction are important pathophysiological events in the occurrence and development of DM complications and cardiovascular events. Masaru Kuroda’s study reported that compared with a marker of averaged blood glucose level such as HbA1c, mean amplitude of glycemic excursions (MAGE) was a more contributing factor of vascular injury [25]. Increasing evidence shows that fluctuant hyperglycemia is more harmful for the heart and vessels compared to persistent hyperglycemia [3, 26], but the specific mechanism remains poorly understood. Previous studies have focused on the influence of PG fluctuation on endothelial cells in vitro [13, 16, 18, 27, 28]; however, few in vivo studies have been conducted. In the present study, an in vivo model of acute fluctuant hyperglycemia was successfully established. In vivo and in vitro experiments have confirmed the effects of continual or intermittent high glucose on endothelial cells are unrelated to the hyperosmotic condition [13, 29]. In A. RISSO’s study [13], Osmotic control was assured by incubating endothelial cells with 20 mmol/l mannitol, both continuously or in alternating fashion. Nonspecific effects due to osmolarity changes had be ruled out, because the cytotoxic effect was not detected in the cells cultured in the presence of 20 mmol/l mannitol, either continuously or in alternating fashion. It demonstrated that the effects of continual or intermittent high glucose are unrelated to the hyperosmotic condition. Our results showed that the 48 h persistent hyperglycemia did not cause apoptosis and dysfunction of vascular endothelial cells in vivo,while acute fluctuant hyperglycemia caused significantly apoptosis. But the Mechanism is not clear, the endothelial cell apoptosis and dysfunction are likely due to oxidative stress and inflammation effects,but may also be secondary to insulin resistance or β-cell dysfunction, and so on. In this study, both the AFG and CHG groups induced insulin resistance, and decreased the β-cell function. However, we observed that neither the HOMA-IR or HOMA-β % showed significant difference between the AFG and CHG group. Moreover, acute fluctuant hyperglycemia could significantly increase oxidative stress and inflammation of vascular endothelial cells in vivo compared to persistent hyperglycemia. So we speculated that, the endothelial cell apoptosis and injury are mainly associated with oxidative stress and inflammatory effects.

Although the impact of hyperglycemia on increasing the oxidative stress of endothelial cells has been confirmed [3, 30–32], few studies have compared the effects of intermittent and chronic hyperglycemia on vascular endothelial cell oxidative stress in vivo. Oxidative stress refers to the production of ROS, overwhelming their clearance and leading to their accumulation. A variety of pathological factors may cause excess production of ROS or reduced clearance of ROS, which disrupts the balance between oxidation and anti-oxidation, increases ROS in the blood and finally results in a oxidative status [33, 34]. Evidence indicates that oxidative stress is a major mechanism of vascular endothelial dysfunction, and ROS production increased significantly in persistent hyperglycemia, accompanied by reduced antioxidase activity, suggesting compromised anti-oxidative activity [3, 30–32]. *In vitro* studies show that fluctuant hyperglycemia may increase oxidative stress of human coronary endothelial cells [16]. Monnier et al. [3] found fluctuant hyperglycemia could also increase oxidative stress in the human body. P47phox is a cytosolic component of the NADPH oxidase, which could be activated or inactivated by the extra signal, thus to be the main enzyme responsible for ROS production. [22]. 3-NT is considered as a marker of peroxynitrite-mediated oxidative stress, which occurs during inflammation. MDA is a classic biomarker of oxidative stress and an aldehyde secondary to ROS-induced lipid peroxidation; its level may reflect the severity of oxidative stress and also may serve as an important pathological indicator [35]. GSH-PX is an important antioxidase, and the content and activity of GSH-PX may directly affect the concentration of ROS in cells [36]. Furthermore, plasma 8-isoprostaglandin represents a useful in vivo oxidative stress marker [23]. In the present study, acute fluctuant hyperglycemia dramatically increased the 3-NT expression,the MDA content,the ratio of membrane-bound to cytosolic portion of p47phox,and the ROS formation, while GSH-PX activity was reduced significantly, suggesting that oxidative stress is a major cause of endothelial injury. Furthermore, acute fluctuant hyperglycemia had a more potent capability to induce oxidative stress as compared to persistent hyperglycemia, which is consistent with previous findings [16].

Studies have shown that hyperglycemia may induce apoptosis of vascular endothelial cells in vitro [13, 16, 37–41]. In addition, transient fluctuant PG may also induce apoptosis of vascular endothelial cells, and more endothelial cells became apoptotic after fluctuant hyperglycemia than after persistent hyperglycemia [13, 14, 16, 39]. However, few studies have been conducted to investigate the influence of acute fluctuant hyperglycemia on apoptosis of endothelial cells in vivo. In the present study, acute fluctuant hyperglycemia significantly increased the number of apoptotic rat vascular endothelial cells and induced endothelial dysfunction; however, endothelial cell apoptosis and dysfunction was absent following persistent hyperglycemia. Furthermore, we detected the expression of the apoptosis-related proteins, Bax,Bcl-2, pro caspase-3 and caspase-3 p17, in endothelial cells. Bcl-2 and Bax are the most important pair of proteins with opposite functions [42]. Mitochondrial translocation of Bax increases mitochondrial membrane permeability and consequently favors the release of apoptogenic factors, such as cytochrome C, leading to the expression of caspase 3 [43]. Caspase-3 is a crucial executive protease in the process of apoptosis [44]. Our results demonstrated that persistent hyperglycemia and fluctuant hyperglycemia significantly increased the expression of Bax protein and promoted its translocation to the outer mitochondrial membrane, and markedly reduced Bcl-2 protein expression, which was accompanied by elevated expression of caspase-3 p17. Of note, as compared to persistent hyperglycemia, fluctuant hyperglycemia increased Bax translocation to mitochondria and caspase-3 p17 expression and reduced Bcl-2 expression to greater levels. Taken together, both acute fluctuant hyperglycemia and persistent hyperglycemia may initiate the apoptosis and dysfunction of endothelial cells, but persistent hyperglycemia failed to significantly induce endothelial apoptosis and dysfunction, which might be ascribed to the short-term exposure to hyperglycemia used in this study. Thus, we speculate that acute fluctuant hyperglycemia is more harmful to endothelial cells than persistent hyperglycemia.

There is a growing body of evidence for the complex involvement of inflammation in DM and its resultant cardiovascular complications [18]. Prabhakaran Kumar et al. [45] demonstrated that high glucose could regulate the expression of IL-6 and IL-17 family cytokines transcriptionally through oxidative stress and NF-κB activation via PKC and p38 MAPK signaling pathways. Finally, high glucose also increases the adhesion of lymphocytes to endothelial surfaces and thereby promoting the pathogenesis of atherosclerosis [45]. Pro-inflammatory cytokines, such as IL-6 and TNF-α, play important roles in the occurrence and development of vascular atherosclerosis [46–48]. It has also been widely accepted that the adhesion of monocytes and macrophages to endothelial cells is crucial for the pathogenesis of atherosclerosis [49]. ICAM-1 is an important adhesion molecule, mediating intercellular adhesion. In vascular endothelial cells, basal ICAM-1 expression is at a low level. ICAM-1 may bind to the specific receptor on vascular endothelial cells to exert its biological effects. In studies using umbilical vein endothelial cells, fluctuant hyperglycemia could significantly increase the content of IL-6, TNF-α and ICAM-1 in vitro [15, 17, 18, 50]. Esposito et al. [51] also found that fluctuant hyperglycemia could induce TNF-α production in the blood of human in vivo. Previous study have shown that high glucose altered the expression profile of cytokines/chemokines via specific signaling pathways and can increase monocyte-endothelial adhesion in monocytes [52]. In our study, an acute increase in PG could increase the plasma levels of IL-6, TNF-α and ICAM-1 and their expression by vascular endothelial cells, and greater inductions were observed following exposure to fluctuant hyperglycemia. Thus, for the first time, our study indicates that acute fluctuant hyperglycemia has a more potent ability to increase pro-inflammatory cytokines production in vivo as compared to persistent hyperglycemia, which is consistent with previous studies in vitro [15, 17, 18, 50]. In summary, our results indicate that acute fluctuant hyperglycemia is likely to induce more severe inflammation and may be more harmful to the cardiovascular system as compared to persistent hyperglycemia.

## Conclusion

Taken together, our results confirm that fluctuant hyperglycemia may significantly increase oxidative stress and inflammation in endothelial cells, elevate the adhesion of monocytes to endothelial cells, increase endothelial cell apoptosis and dysfunction, and cause more severe cardiovascular injury in vivo. It suggests that glucose fluctuation could have a more ominous impact on the function of endothelial cells to promote atherosclerosis. Therefore, not only lowering blood glucose, but also reducing glucose fluctuation is very important in clinic. Based on the control of blood glucose fluctuation, anti-oxidative and anti-inflammatory therapies may provide an alternative for the treatment of fluctuant hyperglycemia-induced cardiovascular diseases.

## Abbreviations

DM: diabetes mellitus
PG: plasma glucose
HCAECs: human coronary artery endothelial cells
SAL: saline
CHG: constant high glucose
AFG: acute blood glucose fluctuation
RIAs: radioimmunoassays
HOMA-IR: homeostasis model of assessment for insulin resistence index
ELISA: enzyme-linked immunosorbent assay
MDA: malondialdehyde
IL-6: interleukin-6
TNF-α: tumor necrosis factor-α
ICAM-1: intercellular adhesion molecule-1
ROS: reactive oxygen species
TUNEL: terminal deoxynucleotidyl transferase dUTP nick end labeling
Ach: acetylcholine
Bcl-2: B-cell lymphoma-2
Bax: B-cell lymphoma-2 associated X protein
3-NT: 3-nitrotyrosine
GSH-PX: glutathione peroxidase
